# Comparing different montages of transcranial direct current stimulation on dual-task walking and cortical activity in chronic stroke: double-blinded randomized controlled trial

**DOI:** 10.1186/s12883-022-02644-y

**Published:** 2022-03-25

**Authors:** Pei-Ling Wong, Yea-Ru Yang, Shun-Chang Tang, Shih-Fong Huang, Ray-Yau Wang

**Affiliations:** 1 Department of Physical Therapy and Assistive Technology, National Yang Ming Chiao Tung University, Taipei, Taiwan, ROC; 2 Division of Nerve Repair- Department of Neurosurgery, Taipei Veterans General Hospital, Taipei, Taiwan, ROC

**Keywords:** tDCS, Dual task, Gait, Contralesional cortical activity, Chronic stroke

## Abstract

**Background:**

Transcranial direct current stimulation (tDCS) is a noninvasive brain stimulation to modulate cortical activity for improving motor function. However, the different tDCS applications for modulating cortical activity and dual task gait performance in chronic stroke have not yet been investigated. This study investigated the effects of different tDCS applications on dual task gait performance and contralesional M1 activation in chronic stroke.

**Methods:**

Forty-eight participants were randomized to anodal, bilateral, cathodal, and sham tDCS groups. Each group received 20 min of tDCS stimulation, except the sham group. Gait performance was measured by GaitRite system during cognitive dual task (CDT) walking, motor dual task (MDT) walking, and single walking (SW). Contralesional M1 activity of unaffected tibialis anterior (TA) was measured using transcranial magnetic stimulation (TMS). Intragroup difference was analyzed by Wilconxon sign ranks test with *Bonferroni correction,* and Kruskal–Wallis one-way analysis of variance by ranks was used for intergroup comparisons, followed by post-hoc Mann–Whitney U tests with Bonferroni correction.

**Results:**

The bilateral tDCS (*p* = 0.017) and cathodal tDCS (*p* = 0.010) improved the CDT walking speed more than sham group. The bilateral tDCS (*p* = 0.048) and cathodal tDCS (*p* = 0.048) also improved the MDT walking speed more than sham group. Furthermore, bilateral tDCS (*p* = 0.012) and cathodal tDCS (*p* = 0.040) increased the silent period (SP) more than the anodal and sham group. Thus, one-session of bilateral and cathodal tDCS improved dual task walking performance paralleled with increasing contralesional corticomotor inhibition in chronic stroke.

**Conclusions:**

Our results indicate that one-session of bilateral and cathodal tDCS increased contralesional corticomotor inhibition and improved dual task gait performance in chronic stroke.

**Trial registration:**

Thai Clinical Trials Registry (TCTR20180116001). Registered prospectively on 16th Jan, 2018 at http://www.thaiclinicaltrials.org.

**Supplementary Information:**

The online version contains supplementary material available at 10.1186/s12883-022-02644-y.

## Background

Most stroke patients demonstrate impaired lower limb movement control due to damaged central neural system which may result in abnormal gait patterns, especially decreased gait speed [1]. Gait speed is considered an important measure of walking ability and it is the most widely used as a primary measure of post-stroke gait performance [2, 3]. However, walking is usually not a single task during daily activities but requires performing another task simultaneously, i.e. the dual task walking. The dual task walking includes cognitive dual task (CDT) walking and motor dual task (MDT) walking. Bowen et al*.* reported that performing a CDT decreased balance and gait speed in stroke patients [2]. Yang et al. noted significant gait decrement during MDT walking in people with stroke as compared with age-matched healthy control [3]. Goh et al., showed that the cognitive and motor tasks resulted in equal decrements in comfortable walking speeds of patients with chronic stroke [4]. Therefore, both CDT and MDT walking are challenging to people with stroke due to additional demand on the already impaired walking ability [5].

Regarding the cortical activity, the decreased ipsilesional hemisphere excitability and increased contralesional hemisphere excitability leading to abnormal interhemispheric interactions were found after stroke [6–8]. The motor dysfunction has been demonstrated to correlate with not only reduced ipsilesional excitability but also the disinhibition of contralesional M1 [8]. Transcranial direct current stimulation (tDCS) is a non-invasive brain stimulation (NIBS) technique of neuromodulation to generate specific changes of the cortical excitability [9]. It has been demonstrated that anodal tDCS can result in neuronal depolarization leading to an increase in excitability. While the cortical excitability is decreased by cathodal stimulation as a result of neurons hyperpolarization [10]. As a result, the tDCS is suggested to modulate cortical activity and thus enhance motor function after stroke. Previous studies indicated that single session of anodal tDCS increased lower extremity muscle strength transiently [11] and a single session of bilateral tDCS improved gait performance [12]. On the other hand, according to recent studies, the single session of tDCS did not exert beneficial effects on gait performance in individuals with chronic stroke [13, 14]. Therefore, the possible effects of tDCS need to be further investigated. Regarding cortical activities, only one study reported the cathodal tDCS applying over the contralesional motor cortex could enhance the motor training effects of paretic hand by modulating cortical excitability [15]. However, to our knowledge, there was no study investigating the different effects of tDCS electrode arrangements on dual task walking performance and cortical modulation in people with chronic stroke. Thus, the purpose of present study was to compare the effects of different tDCS montages on dual task gait performance and cortical activity in chronic stroke.

## Methods

### Subjects

The study protocol was approved by the Institutional Review Board of Taipei Veterans General Hospital and National Yang-Ming University. This trial was registered at http://www.thaiclinicaltrials.org (TCTR20180116001 on 16/01/2018) and conformed to the CONSORT checklist. All experiments were performed in accordance with relevant guidelines and regulations. Participants with chronic stroke were recruited from a medical center between March 2018 and July 2019. Stroke diagnosis, age, gender, stroke type, lesion site, and poststroke duration were obtained from a detail clinical interviews and medical charts. Inclusion criteria were: (1) 6 months post first-ever stroke with unilateral motor deficits, (2) ability to walk independently for at least 10 m without using walking aids, and (3) a score of ≥ 24 on the mini-mental state examination (MMSE). Exclusion criteria were (1) unstable medical conditions, and (2) history of other diseases or conditions known to interfere with participating the study (e.g., epilepsy or metal implants in the brain). All participants provided signed informed consent before participation.

### Experimental design

**Fig. 1:**
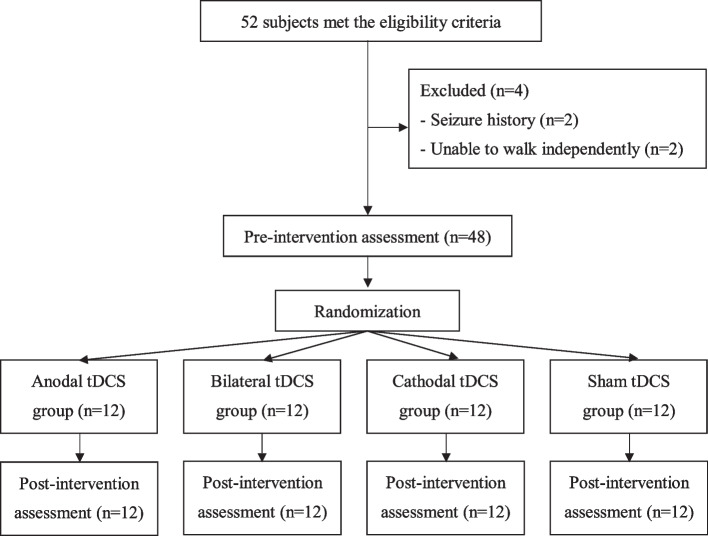
Flowchart of the patient inclusion and study procedures (*n* = 48)

This study was a double-blinded, randomized, controlled trial with pre- and post- measurements. An individual who was not involved with the study selected sealed envelopes to assign participants to one of the four groups: anodal tDCS group, cathodal tDCS group, bilateral tDCS group, and sham tDCS group. Participants were blinded to their group assignment (participants blinded). In this study, participants received one session of real or sham tDCS for 20 min according to their group assignment. The outcomes included the gait performance and brain activities measured on the same day before (pre-test) and immediately after (post-test) the real (or sham) tDCS by the assessor who was blinded to the group assignment (assessor blinded) (Fig. 1).

### Intervention

The tDCS protocol in this study followed the protocol described by Mahmoudi et al. [16]. The stimulation delivered by a current stimulator *(Eldith DC Stimulator, NeuroConn, Germany)* through a pair of 35 cm^2^ electrodes with a maximal output of 2 mA. The stimulation intensity was set to 2 mA for 20 min with a current density of 0.07 C/cm^2^ which is well below the threshold for tissue damage [17]. The placement of electrodes for different groups were described in the following [17]:

- Anodal tDCS group: The anode was placed over the ipsilesional M1 (primary motor cortex, C3 or C4 according to EEG 10/20 system), and the cathode over the contralateral supraorbital ridge.
- Cathodal tDCS group: The cathode was placed over the contralesional M1, and the anode over the contralateral supraorbital ridge.
- Bilateral tDCS group: The anode was placed over ipsilesional M1 and the cathode over contralesional M1.
- Sham tDCS group: The electrodes were positioned as described in the anodal tDCS group. However, the current was only delivered for 60 s, with a ramp up and ramp down for 30 s [18].

### Outcome measures

The primary outcome was speed during CDT walking MDT walking. The secondary outcomes included temporal–spatial gait parameters during CDT and MDT walking and single walking, corticomotor activity, and lower extremity motor performance.

### Gait performance

The gait performance under 3 different conditions: CDT walking, MDT walking, and single walking was measured by a GAITRite system (CIR system, Inc., Havertown, Pennsylvania). Each walking condition was repeated twice, and the sequence of total six walking conditions was randomized with 60 s rest between each walking condition. The average of the two trials of each walking condition was used for data analysis. The CDT walking was walking while serial subtracting by three, starting from a randomized 3-digit number (e.g. 211, 208, 205…) at comfortable speed. The MDT walking was walking while carrying a tray with a bottle of water in front of the subject with the non-affected hand at comfortable speed. For single walking performance, participants walked with their comfortable speed without additional task. The GAITRite system is a straight walkway containing pressure-sensitive sensors. The walkway is 4.75 m long and 0.9 m wide, and the pressure-sensitive area is 4.30 m long and 0.61 m wide. When participants walk along the walkway, the contact time and location of each footfall are recorded and analyzed using the application software. The concurrent validity has been established and the test–retest reliability while executing dual tasks in stroke subjects has been proven [19, 20].

Other gait parameters as secondary outcomes included cadence, step time, step length, and coefficients of variation (CV) of step time and step length. The formula of CV is standard deviation/mean × 100%. A lower value indicates a more consistent gait pattern. In addition, the dual task cost of gait speed (DTC-speed) was calculated in this study to indicate the dual task interference. The DTC-speed = (dual task walking speed–single task walking speed)/single task walking speed × 100% [21].

### Corticomotor activity

It has been suggested that increased inhibition from contralesional hemisphere may cause abnormal interhemispheric activation to possibly result in impaired motor performance in stroke [22]. Therefore, in this study, we measured the corticomotor activity of contralesional hemisphere to explore the modulating effects of different tDCS placements in parallel with gait performance. The resting motor threshold (RMT), silent period (SP) duration, and short interval intracortical inhibition (SICI) of the unaffected tibialis anterior (TA) muscle elicited by transcranial magnetic stimulation (TMS) were used to indicate the corticomotor excitability.

The motor evoked potentials (MEPs) of the tibialis anterior muscles were recorded by an electromyographic (EMG) machine in response to TMS (Magstim 200 magnetic stimulator, Magstim Company, Whiteland, Dyfed, UK) delivered through a double-cone coil placed on the contralesional M1 with the participants lying supine comfortably wearing a fitted cap marked with a coordinate system (distance, 1 cm). Muscle activity was carefully monitored by real-time EMG. The coil was positioned parallel with the transverse plane and the handle of the coil pointed outward to induce a lateral-to-medial current flow in brain. The optimal scalp location (hot spot) was determined by moving the TMS stimulator over the scalp in 1-cm steps. Once the hot spot was identified, a single-pulse TMS was delivered to the location to determine the RMT, defined as the lowest stimulus intensity necessary to elicit MEPs greater than 0.05-mV peak-to-peak amplitude in at least 5 of 10 consecutive stimuli [23]. The RMT was expressed as a percentage of maximum stimulator output. The SP duration was determined during isometric voluntary contraction of tibialis anterior muscle. Ten magnetic stimuli were applied at an intensity of 120% RMT while the participant performed maximum 20% voluntary contraction. The SP duration was determined from the MEP onset to the recurrence of at least 50% of EMG background activity [24]. The SP originates largely from activation of inhibitory interneurons. This neurophysiological phenomenon is thought to be due to inhibition mechanisms of the motor cortex mediated through the GABA_B-_ergic system [25]. Paired-pulse paradigm was performed in the relaxed tibialis anterior muscle to assess the SICI. The intensity of the conditioning stimulus was set at 80% RMT with short interstimulus interval (2 ms) and then the testing stimulus at 120% RMT. The SICI was expressed as the percentage of inhibition using the following formula:100 – (conditioned MEP/ unconditioned MEP) x 100 [26]. The SICI is suggested be at least partially GABA_A_ receptor mediated [27]. For each condition, 10 trials were collected and averaged. The intensity used in the post-assessment was the same as that used in the pre-assessment.

### Lower extremity motor performance

Motor control of the lower limb was assessed by Fugl-Meyer assessment (FMA) which was reported to have good reliability for stroke patients [28]. Each item is scored using a 3-point ordinal scale, from 0 (no performance) to 2 (complete performance), with a maximum of 34 points [29]. Higher score indicates better control of the lower extremity.

### Sample size

We calculated the sample size using G*power v3.1.0. According to Tahtis et al.'s study, the effect size was 0.609 for single session tDCS in improving gait speed after stroke [12]. Therefore, we set the effect size of 0.5 for dual task gait improvement, power of 0.80 and a two-tailed alpha level of 0.05 in present study. The total sample size was required to be 40 (10 per group). To considerate possible dropping-out and missing data, we thus recruited 48 participants.

### Statistical analysis

All analyses were performed using the SPSS 24.0. Descriptive statistics (mean± standard deviation) were generated for all variables. Normal distribution of outcomes cannot be confirmed by Shapiro-Wilk test, and thus intergroup differences among baseline characteristics and pre-test measures were evaluated using Kruskal-Wallis one-way analysis of variance by ranks or χ2 analysis. Intragroup difference was analyzed by Wilconxon sign ranks test with Bonferroni correction to determine the changes after tDCS. To compare the intergroup differences, the change values were analyzed using Kruskal-Wallis one-way analysis of variance by ranks, followed by post-hoc Mann-Whitney U tests with Bonferroni correction. Additionally, due to 11 parameters were measured during gait performance, we corrected the p-values of Kruskal-Wallis test for each gait parameter using Bonferroni correction which multiplied the uncorrected p-values by 11. Change values were calculated by subtracting the base-line data from the post-intervention data. Statistical significance was set at p<0.05. The Z/√N and η^2^_H_=H-k+1/n-k were calculated to indicate the effect size (ES) (r) of intragroup and ES (η^*2*^) of intergroup[30]. ES(r) greater than 0.5 represents large, 0.3 indicates medium and 0.1 indicates small intragroup ES. For intergroup comparison, ES(η^*2*^) greater than 0.14 indicates large, 0.06 indicates medium and 0.01 indicates small intergroup ES [31].

## Results

A total of 52 patients were screened for the eligibility of participating the study. As a result, 48 participants were included and were randomized to the anodal tDCS group (*n *= 12), bilateral tDCS group (*n *= 12), cathodal tDCS group (*n *= 12), or sham tDCS group (*n *= 12). Participants received 20 min of tDCS according to their group assignment. None of them reported any adverse events or withdrew from the study (Fig. 1). No significant differences between groups were found for baseline demographic characteristics (Table 1). Similarly, no significant differences between groups were found for any of the outcome measures at the pre-intervention assessment.

**Table 1:** Demographic characteristics of included stroke participants (*n* = 48)

	**Anodal group (***n*** = 12)**	**Bilateral group (***n*** = 12)**	**Cathodal group (***n*** = 12)**	**Sham group (***n*** = 12)**	***P* value**
Age (years)	52.7 (45.3, 64.5)	51.8 (42.5, 65.1)	59.1 (51.6, 66.8)	57.3 (46.1, 62.8)	0.72
Gender (Male / Female)	10/2	9/3	10/2	9/3	0.92
Type of stroke (ischemic/hemorrhagic)	6/6	8/4	7/5	4/8	0.40
Location of lesion (C/S/M)	1/10/1	2/10/0	0/11/1	1/10/1	0.80
Lesion side (Left/Right)	3/9	3/9	8/4	8/4	0.07
Post stroke period (months)	74.4 (20.4, 88.7)	66.6 (37.3, 86.0)	45.1 (20.0, 105.0)	54.0 (24.0, 93.4)	0.97
MMSE	29.0 (28.3, 30.0)	29.00 (27.5, 29.8)	29.0 (28.0, 30.0)	28.0 (26.3, 29.0)	0.42

Data are presented as the median (interquartile range) or number

*Abbreviations*: *MMSE* mini-mental state examination

C/S/M indicates cortical, subcortical, and mixed cortical & subcortical

### Dual task walking performance

**Fig. 2:**
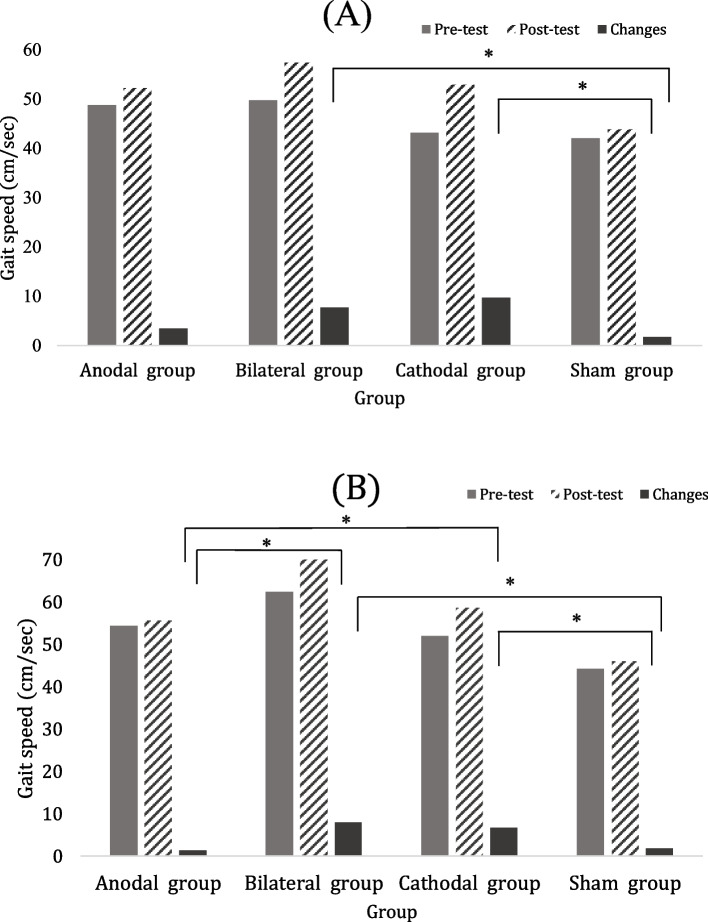
Change in gait speed during dual task walking performance after different tDCS stimulations. **A** cognitive dual task (CDT) walking. **B** motor dual task (MDT) walking (^*^ *p* < 0.05 intergroup comparison)

Table 2 shows the CDT walking performance at pre- and post-intervention for 4 studied groups. The improvements in gait speed were significant after bilateral *(p*=*0.012, ES*_*(r)*_=*0.43 )* and cathodal *(p*=*0.012 , ES*_*(r)*_=*0.43 )* tDCS, and such improvements were significantly more than the sham group (Fig. 2A) *(bilateral vs. sham group, p*=*0.017, ES*_*(*_η^*2*^_*)*_ =*0.32; cathodal vs. sham group, p*=*0.010, ES*_*(*_η^*2*^_*)*_ =*0.34)*. The increased step length of unaffected leg were noted after bilateral tDCS(*p*=*0.036, ES*_*(r)*_ =*0.38)* and cathodal tDCS (*p*=*0.032, ES*_*(r)*_ =*0.38*). However, the increased cadence was only found after bilateral tDCS *(p*=*0.016, ES*_*(r)*_=*0.42)*.

Table 3 shows the MDT walking performance after tDCS applications. The increased gait speed *(p*=*0.02, ES*_*(r)*_=*0.41)* was demonstrated after bilateral tDCS which was significantly more than the anodal tDCS *(p*=*0.018, ES*_*(*_η^*2*^_*)*_ =*0.45)* and the sham tDCS *(p*=*0.048, ES*_*(*_η^*2*^_*)*_=*0.28).* After cathodal tDCS, the MDT walking speed also increased, *(p*=*0.012, ES*_*(r)*_=*0.43)* and such increase was significantly more than the anodal tDCS *(p*=*0.018, ES*_*(*_η^*2*^_*)*_=*0.45)* and the sham tDCS *(p*=*0.048, ES*_*(*_η^*2*^_*)*_ =*0.28)* (Fig. 2B).

**Table 2:** Cognitive dual task (CDT) walking performance after different tDCS interventions (*n* = 48)

**Group**	**Anodal group (***n*** = 12)**		**Bilateral group (***n*** = 12)**		**Cathodal group (***n*** = 12)**		**Sham group (***n*** = 12)**		
	pre	post	pre	post	pre	post	pre	post	p value*
Speed_(cm/sec)_	43.3 (34.5, 77.5)	52.4 (37.8, 78.5)	55.2 (33.9, 61.3)	64.4 (39.7, 70.9)^#^	45.6 (29.2, 55.7)	51.3 (41.8, 64.5)^#^	39.5 (26.2, 57.2)	39.6 (28.8, 57.9)	
change	3.7 (0.5, 6.2)		8.3 (3.2, 11.3)^s^		6.7 (4.7, 12.6)^s^		1.0 (0.2, 4.0)		0.033
Cadence_(step/min)_	82.9(63.1, 105.5)	82.6 (67.0, 100.7)	78.3 (60.7, 88.1)	84.0 (62.7, 95.8)^#^	68.9 (55.0, 81.0)	78.3 (72.0, 88.2)	77.2 (61.5, 87.5)	79.5 (66.0, 88.5)	
change	0.7(-4.7, 3.3)		6.4 (2.8, 10.0)		5.9(-0.9, 19.4)		1.9 (0.9, 4.2)		NS
Step time _(UA)(sec)_	0.7 (0.5, 0.8)	0.6 (0.5, 0.7)	0.6 (0.6, 0.8)	0.6 (0.6, 0.7)	0.7 (0.6, 0.8)	0.7 (0.6,0.7)	0.7 (0.6, 0.8)	0.7 (0.6, 0.8)	
change	0.0 (0.0, 01)		0.0 (-0.1, 0.0)		0.0 (-0.1, 0.0)		0.0 (-0.1, 0.0)		NS
Step time_(A)(sec)_	0.8 (0.6, 1.1)	0.8 (0.6, 1.1)	0.9 (0.8, 1.3)	0.8 (0.7, 1.1)	1.0 (0.8, 1.2)	0.9 (0.8, 1.0)	0.9 (0.7, 1.0)	0.8 (0.9, 0.7)	
change	0.0 (-0.1, 0.1)		-0.1 (-0.1,0.0)		-0.1(-0.3, 0.0)		-0.1 (-0.1, 0.0)		NS
Step length _(UA)(cm)_	34.5 (22.1, 46.0)	35.9 (22.9, 46.7)	33.8 (31.3, 41.2)	38.7 (35.2, 41.9)^#^	38.3 (29.2, 46.1)	41.8 (29.9, 49.8)^#^	31.9 (22.5, 37.7)	31.9 (21.6, 36.0)	
change	2.3 (0.4, 4.9)		1.9 (1.3, 4.9)		3.0 (0.6, 4.4)		-0.6 (-2.7, 1.2)		NS
Step length_(A)(cm)_	40.0 (27.0, 48.2)	40.9 (28.3, 51.5)	43.9 (37.6, 48.9)	45.3 (41.0, 55.4)	38.5 (30.7, 44.6)	41.2 (34.1, 46.5)	33.6 (22.7, 44.6)	34.2 (22.5, 37.5)	
change	1.5 (0.0, 4.3)		2.9 (1.5, 5.8)		3.5(2.2, 6.6)		0.6 (-1.4, 3.6)		NS
ST_(UA)_ variability _(%)_	10.1 (3.9, 20.4)	7.1 (4.2, 23.8)	8.62 (5.5, 39.1)	6.2 (3.2, 11.9)	8.5 (6.0, 12.4)	7.1 (4.5, 10.8)	6.6 (3.4, 8.9)	6.7 (4.9, 11.0)	
change	1.0 (-5.9, 5.4)		-2.8(-21.1, -1.0)		-1.6 (-4.9, 4.2)		0.9 (-1.1, 3.7)		NS
ST_(A)_ variability _(%)_	6.3 (4.0, 9.7)	5.6 (3.1, 12.3)	8.2 (6.1, 23.0)	5.7 (3.9, 13.3)	6.1 (4.8, 18.1)	6.6 (4.0, 11.9)	6.2 (2.7, 12.0)	4.9 (4.2, 7.8)	
change	-1.7 (-3.2, 2.6)		-3.4(-8.7, -0.6)		-0.5 (-9.3, 2.0)		-0.3 (-3.5, 3.7)		NS
SL_(UA)_ variability _(%)_	8.4 (2.9, 15.7)	5.8 (3.2, 13.4)	7.9 (4.7, 12.3)	4.9 (4.4, 12.2)	8.3 (4.8, 14.4)	6.5 (4.6, 9.4)	7.3 (5.4, 12.3)	8.5 (5.4, 11.2)	
change	1.1 (-4.6, 5.2)		-1.8 (-3.2, 2.9)		-2.1 (-6.0, 0.8)		0.6 (-1.5, 3.6)		NS
SL_(A)_ variability _(%)_	7.1 (4.6, 13.0)	6.5 (4.0, 9.0)	7.6 (5.0, 13.6)	4.7 (3.3, 9.5)	6.6 (5.3, 8.2)	8.0 (4.6, 9.4)	8.4 (5.1, 26.8)	6.1 (4.3, 11.0)	
change	-0.7 (-6.8, 1.3)		-2.8 (-5.8, 1.1)		-0.2 (-2.2, 2.9)		-3.5 (-10.6, 1.0)		NS
DTC _(%)_	-22.3(-34.2, -7.0)	-8.0 (-24.2, -4.3)	-16.6(-51.6, -7.4)	-24.8(-40.7, -10.9)	-15.0(-41.8, -5.2)	-15.9 (-26.6, -9.4)	-19.5(-32.3,-9.1)	-21.8(-29.4, -8.4)	
change	10.2 (-3.0, 18.8)		0.9 (-10.2, 11.9)		-0.1 (-5.4, 19.0)		2.5 (-3.5, 13.0)		NS

Data are presented as the median (interquartile range)

Abbreviations: UA, unaffected side; A, affected side; ST, step time; SL, step length; DTC, dual task cost. NS, Not significant (*P* > 0.05)

^*^P value based on Kruskal–Wallis one-way analysis of variance by ranks with Bonferroni *correction*

^#^, *p* < 0.05 for intragroup comparison. ^a^, *p* < .05 as compared with Anodal group. ^s^, *p* < .05 as compared with Sham group

**Table 3:** Motor dual task (MDT) walking performance after different tDCS interventions (*n* = 48)

**Group**	Anodal group (*n* = 12)		Bilateral group (*n* = 12)		Cathodal group (*n* = 12)		Sham group (*n* = 12)		
	pre	post	pre	post	pre	post	pre	post	p value*
Speed_(cm/sec)_	52.0 (38.6, 77.5)	51.7 (45.3, 76.2)	50.3 (40.3, 79.4)	61.2 (52.8, 82.7)^#^	52.1 (38.7, 63.1)	57.1 (50.2, 69.3)^#^	42.4 (27.7, 60.1)	40.8 (27.0, 63.2)	
change	1.6 (-2.2, 2.9)		7.0 (4.5, 11.6)^a,s^		6.7 (4.2, 8.9)^a,s^		0.9 (-1.0, 3.3)		0.011
Cadence_(step/min)_	96.1(82.4, 112.0)	97.3 (81.8, 111.3)	84.2 (77.4, 98.3)	89.1(83.6, 101.1)^#^	83.9 (73.5, 98.6)	88.3 (82.0, 98.2)	87.5 (63.9, 97.5)	84.0 (61.8, 95.2)	
change	0.1 (-5.1, 3.0)		3.5 (0.4, 10.0)		4.6 (-0.6, 7.6)		-1.5 (-4.5, 4.4)		NS
Step time _(UA)(sec)_	0.6 (0.5, 0.6)	0.6 (0.5, 0.7)	0.6 (0.5, 0.7)	0.6 (0.5, 0.6)	0.6 (0.5, 0.7)	0.6 (0.5,0.7)	0.6 (0.5, 0.7)	0.6 (0.5, 0.8)	
change	0.0 (0.0, 0.0)		0.0 (-0.1, 0.0)		0.0 (-0.1, 0.0)		0.0 (0.0, 0.0)		NS
Step time_(A)(sec)_	0.7 (0.6, 0.9)	0.8 (0.6, 0.9)	0.9 (0.6, 1.0)	0.8 (0.6, 0.9)^#^	0.9 (0.7, 0.9)	0.8 (0.8, 0.9)	0.8 (0.7, 1.0)	0.8 (0.7, 1.1)	
change	0.0 (0.0, 0.1)		0.0 (-0.1, 0.0)		0.0 (-0.1, 0.0)		0.0 (0.0, 1.0)		NS
Step length _(UA)(cm)_	37.2 (25.9, 39.7)	36.7 (25.1, 40.1)	36.2 (25.6, 49.4)	39.6 (29.1, 49.9)	37.2 (25.3, 45.8)	38.5 (30.3, 47.2)	27.5 (21.8, 32.0)	28.2 (21.2, 35.9)	
change	1.0 (-3.4, 2.9)		1.1 (-0.4, 6.1)		1.8 (-0.8, 4.5)		0.0 (-2.2, 4.2)		NS
Step length_(A)(cm)_	36.6 (28.5, 47.7)	39.4 (30.6, 53.6)	43.7 (34.5, 54.0)	46.8 (43.1, 56.3)	38.0 (31.0, 42.2)	41.8 (34.1, 46.2)^#^	31.7 (28.6, 40.9)	33.2 (27.1, 39.9)	
change	1.8 (-0.5, 6.2)		1.6 (0.1, 4.3)		3.1 (1.6, 4.8)		2.5 (-1.3, 4.2)		NS
ST_(UA)_ variability _(%)_	7.6 (5.0, 10.7)	3.9 (3.2, 5.8)	8.0 (3.0, 10.6)	8.1 (1.9, 8.4)	7.6 (6.5, 8.8)	5.8 (2.5, 9.3)	4.7 (3.4, 9.4)	6.5 (4.0, 8.2)	
change	-2.6 (-4.6, -0.8)		0.0(-4.2, 3.9)		-1.7 (-3.5, 0.7)		0.5 (-4.0, 2.7)		NS
ST_(A)_ variability _(%)_	5.4 (3.4, 8.0)	3.9 (3.1, 6.7)	4.7 (2.3, 10.8)	5.1 (1.9, 8.4)	7.4 (3.3, 10.0)	6.5 (3.7, 7.7)	4.7 (3.6, 6.1)	5.7 (3.7, 8.6)	
change	-1.0 (-2.4, 1.4)		0.1(-3.3, 2.4)		-1.2 (-2.4, 1.4)		1.4 (-0.2, 3.6)		NS
SL_(UA)_ variability _(%)_	8.2 (5.0, 14.8)	7.1 (4.8, 18.2)	9.8 (5.7, 15.2)	6.9 (4.7, 15.2)	9.0 (3.6, 14.4)	6.9 (3.8, 11.1)	9.9 (6.5, 15.1)	8.4 (4.8, 19.1)	
change	-0.2 (-4.6, 3.8)		-0.6 (-3.3, 2.1)		-0.1 (-5.5, 1.8)		-1.1 (-3.7, 5.5)		NS
SL_(A)_ variability _(%)_	5.2 (4.3, 11.5)	6.9 (5.4, 8.5)	6.2 (2.8, 10.4)	4.7 (4.1, 9.5)	5.6 (3.4, 7.9)	5.3 (4.4, 6.8)	5.9 (4.4, 9.8)	6.5 (4.2, 13.6)	
change	-0.7 (-3.3, 2.4)		0.4 (-2.0, 1.8)		-0.3 (-3.3, 1.6)		0.3 (-3.6, 3.4)		NS
DTC _(%)_	-7.2(-18.2, -0.8)	-9.0 (-14.8, 1.6)	-10.2(-29.7, -2.2)	-9.8 (-25.8, -4.1)	-14.1(-20.7, -0.3)	-18.5 (-24.1, -1.7)	-10.2(-25.5, 7.2)	-13.8 (-21.7, 0.5)	
change	3.1 (-3.5, 8.1)		3.6 (-2.1, 9.7)		2.6 (-9.2, 14.4)		-1.5 (-3.8, 6.2)		NS

Data are presented as the median (interquartile range)

*Abbreviations*: *UA* unaffected side, *A* affected side, *ST* step time, *SL *step length, *DTC* dual task cost, *NS* Not significant (*P* > 0.05)

^*^P value based on Kruskal–Wallis one-way analysis of variance by ranks with Bonferroni *correction*

^#^, *p* < 0.05 for intragroup comparison. ^a^, *p* < .05 as compared with Anodal group. ^s^, *p* < .05 as compared with Sham group

### Single walking performance

Table 4 shows the single walking performance after different tDCS interventions. The gait speed increased *(p*=*0.016, ES*_*(r)*_=*0.42)* with increased cadence *(p*=*0.016, ES*_*(r)*_=*0.42)* and decreased step time of both legs *(*affected leg*: p*=*0.016, ES*_*(r)*_=*0.41*/ unaffected leg: *p*=*0.048, ES*_*(r)*_=*0.36)* after bilateral tDCS. The increased in gait speed *(p*=*0.023, ES*_*(*_η^*2*^_*)*_=*0.31)* and cadence *(p*=*0.012, ES*_*(*_η^*2*^_*)*_=*0.33)* were significantly more than the sham tDCS*.* The increased in gait speed (*p*=*0.015, ES*_*(*_η^*2*^_*)*_=*0.46)* was significantly more than the anodal tDCS*.* The increased in cadence *(p*=*0.048, ES*_*(r)*_=*0.36)* and decreased step time of affected leg *(p*=*0.036, ES*_*(r)*_=*0.38)* noted after cathodal tDCS*.* The CDT walking, MDT walking, and single walking performance did not change significantly after anodal tDCS or sham tDCS.

**Table 4:** Single walking (SW) performance after different tDCS interventions (*n* = 48)

**Group**	**Anodal group (*n* = 12)**		**Bilateral group (*n* = 12)**		**Cathodal group (*n* = 12)**		**Sham group (*n* = 12)**		
	pre	post	pre	post	pre	post	pre	post	p value*
Speed_(cm/sec)_	64.23 (50.6, 84.8)	62.7 (48.8, 80.6)	62.4 (52.2, 71.0)	68.8 (64.2, 75.4)^#^	63.8 (52.8, 70.8)	65.4 (57.8, 79.3)	51.6 (41.4, 71.4)	51.8 (42.3, 70.3)	
change	-0.2 (-3.1, 1.9)		5.1 (2.5, 13.0)^a,s^		7.2 (-1.2, 11.6)		0.4 (-2.2, 1.8)		0.030
Cadence_(step/min)_	95.9 (74.8, 106.0)	96.6(78.1, 102.4)	85.7 (80.1, 91.3)	92.0 (87.5, 96.9)^#^	87.5 (74.5, 97.0)	92.5 (81.8,100.6)^#^	87.5 (77.2, 100.5)	85.1 (78.1, 98.6)	
change	-0.1 (-2.6, 4.0)		6.7 (3.8, 10.1)^s^		5.2 (1.1, 9.5)		-0.4 (-2.3, 1.0)		0.030
Step time _(UA)(sec)_	0.6 (0.5, 0.7)	0.6 (0.5, 0.7)	0.6 (0.5, 0.7)	0.5 (0.5, 0.6) ^#^	0.6 (0.6, 0.7)	0.6 (0.5, 0.6)	0.6 (0.5, 0.7)	0.6 (0.5, 0.6)	
change	0.0 (0.0, 0.1)		-0.1 (-0.2, 0.0)		0.0 (-0.1, 0.0)		0.0 (0.0, 0.0)		NS
Step time_(A)(sec)_	0.7 (0.6, 1.0)	0.7 (0.6, 0.9)	0.8 (0.8, 0.9)	0.8 (0.7, 0.9)^#^	0.8 (0.7, 1.0)	0.7 (0.7, 0.9)^#^	0.8 (0.7, 0.9)	0.8 (0.7, 0.9)	
change	0.0 (-0.1, 0.0)		0.0 (-0.1, 0.0)		-0.1 (-0.1, 0.0)		0.0 (0.0, 0.0)		NS
Step length _(UA)(cm)_	40.8 (35.6, 45.5)	41.4 (34.6, 47.1)	41.6 (35.2, 44.7)	40.4 (36.7, 44.8)	43.1 (30.6, 45.6)	43.1 (34.4, 48.7)	30.4 (27.7, 37.8)	34.2 (27.1, 36.1)	
change	2.2 (-2.8, 4.5)		1.1 (-0.4, 2.2)		0.7 (-1.8, 3.5)		0.4 (-1.3, 3.0)		NS
Step length_(A)(cm)_	48.2 (33.9, 57.6)	41.1 (31.2, 51.6)	46.3 (36.8, 53.3)	54.2 (44.4, 57.7)	44.9 (39.1, 47.0)	46.1 (44.2, 49.3)	37.6 (33.0, 46.7)	38.3 (34.0, 47.2)	
change	-1.5 (-5.3, -1.0)		2.0 (-0.8, 7.2)		2.0 (0.1, 5.1)		0.3 (-0.5, 1.7)		NS
ST _(UA)_ variability _(%)_	7.3 (2.9, 11.0)	7.1 (3.7, 12.6)	4.3 (2.8, 7.2)	4.8 (2.8, 5.8)	5.6 (3.4, 7.4)	5.0 (3.8, 6.5)	4.8 (4.6, 7.1)	6.2 (3.4, 8.3)	
change	0.0 (-4.5, 5.8)		1.1 (-0.2, 2.5)		0.1 (-2.1, 1.6)		1.0 (-0.7, 1.8)		NS
ST_(A)_ variability _(%)_	4.0 (2.1, 6.1)	6.0 (3.9, 6.4)	4.9 (3.4, 8.1)	6.0 (4.2, 11.4)	7.0 (3.0, 10.6)	5.0 (3.2, 6.1)	4.4 (3.4, 6.7)	4.3 (3.4, 5.3)	
change	2.2 (-0.7, 5.2)		0.6 (-1.0, 4.4)		-2.6 (-5.7, 1.2)		-1.3 (-3.7, 1.8)		NS
SL _(UA)_ variability _(%)_	5.5 (4.2, 13.4)	8.8 (4.1, 14.8)	5.0 (3.6, 7.7)	6.4 (4.3, 8.5)	4.2 (1.9, 7.9)	6.4 (5.2, 7.8)	7.6 (3.8, 11.7)	9.3 (6.5, 12.6)	
change	2.7 (-1.9, 5.3)		1.6 (-0.8, 2.2)		1.9 (-0.8, 4.4)		1.7 (-1.6, 2.3)		NS
SL_(A)_ variability _(%)_	4.4 (2.2, 6.9)	6.3 (5.0, 7.5)	4.3 (1.7, 6.5)	3.8 (2.2, 6.8)	4.6 (1.8, 9.1)	4.6 (2.2, 7.9)	4.9 (4.4, 8.1)	5.7 (2.3, 6.6)	
change	1.1 (-0.7, 4.2)		0.6 (-1.7, 1.7)		0.4 (-2.6, 2.7)		0.5 (-2.9, 2.4)		NS

Data are presented as the median (interquartile range)

Abbreviations: UA, unaffected side; A, affected side; ST, step time; SL, step length. NS, Not significant (*P* > 0.05)

^*^P value based on Kruskal–Wallis one-way analysis of variance by ranks with Bonferroni *correction*

^#^, *p* < 0.05 for intragroup comparison

^a^, *p* < .05 as compared with Anodal group. ^s^, *p* < .05 as compared with Sham group

### Corticomotor activity

**Fig. 3:**
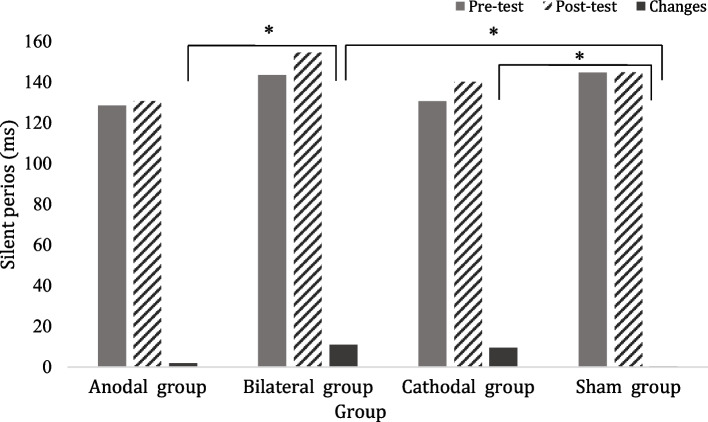
Change in silent period (SP) after different tDCS stimulations (^*^ *p* < 0.05 intergroup comparison)

Table 5 shows the corticomotor activity of contralesional hemisphere measured by the TMS before and after tDCS interventions. The SP increased significantly after bilateral tDCS (*p*=*0.012, ES*_*(*_η^*2*^_*)*_=*0.33*)and cathodal tDCS(*p*=*0.040, ES*_*(*_η^*2*^_*)*_=*0.28*), which was significantly more than sham tDCS (Fig. 3)*.* Furthermore, after bilateral tDCS stimulation, the increased in SP were significantly more than the anodal tDCS *(p*=*0.048, ES*_*(*_η^*2*^_*)*_=*0.41).* However, the anodal and sham tDCS did not change the corticomotor activity.

**Table 5:** Corticomotor activity after different tDCS interventions (*n* = 48)

**Grou*p***	**Anodal group (*n* = 12)**		**Bilateral group (*n* = 12)**		**Cathodal group (*n* = 12)**		**Sham group (*n* = 12)**		
	pre	post	pre	post	pre	post	pre	post	*p value*
RMT _(%)_	58.0 (49.8, 63.8)	58.0 (50.5, 64.5)	63.0 (48.3, 69.5)	63.0 (48.5, 68.8)	62.5 (54.0, 65.8)	65.0 (53.5, 67.8)	57.0 (52.5, 65.5)	57.5 (52.5, 64.0)	
change	0.0 (0.0, 2.0)		1.5 (0.0, 2.8)		2.0 (0.0, 3.0)		0.0 (0.0, 1.0)		0.153
SP (ms)	130.1 (110.2, 139.5)	124.1 (117.4, 136.8)	142.8 (117.2, 168.9)	154.5 (128.3, 189.8)	127.9 (118.8, 144.1)	139.4 (127.3, 156.4)^#^	144.7 (131.4, 156.5)	145.5 (130.4, 156.3)	
change	-1.1 (-3.0, 6.4)		9.2 (3.0, 20.5)^a,s^		7.0 (3.4, 14.6)^s^		1.5 (-3.7, 3.6)		0.002
SICI _(%)_	49.1 (40.9, 58.6)	45.3 (34.3, 60.4)	46.0 (39.2, 65.7)	47.3 (37.7, 58.1)	42.2 (39.6, 48.9)	42.6 (34.4, 48.2)	42.4 (37.2, 63.4)	47.9 (38.2, 59.2)	
change	-2.5 (-6.2, -0.1)		-1.6 (-11.9, 12.0)		1.2 (-9.0, 4.9)		2.8 (-1.7, 7.8)		0.296

Data are presented as the median (interquartile range)

Abbreviations: RMT, resting motor threshold; SP, silent period; SICI, short interval intracortical inhibition

^#^, *p* < 0.05 for intragroup comparison. ^a^, *p* < .05 as compared with Anodal group. ^s^, *p* < .05 as compared with Sham group

### Effects of tDCS on lower limb motor function

The results of FMA after tDCS are shown in Table 6. The scores of FMA were improved after bilateral tDCS *(p*=*0.044, ES*_*(r)*_=*0.37)* and cathodal tDCS *(p*=*0.040, ES*_*(r)*_=*0.37)*. However, there was no significant difference between groups.

**Table 6:** Fugl-Meyer score after different tDCS interventions (*n *= 48)

Group	Anodal group (***n*** = 12)		Bilateral group (***n*** = 12)		Cathodal group (***n*** = 12)		Sham group (***n ***= 12)		
	pre	post	pre	post	pre	post	pre	post	*p value*
FMA	24.0 (19.5, 27.0)	25.0 (20.5, 27.5)	27.0(24.0, 31.0)	27.0(25.0, 31.5)^#^	24.0 (21.0, 26.0)	24.5 (22.0, 27.0)^#^	27.0 (22.5, 28.0)	27.0 (22.5, 28.0)	
change	0.0 (0.0, 1.0)		1.0 (0.0, 1.0)		1.0 (0.0, 1.8)		0.0 (0.0, 0.8)		0.181

Data are presented as the median (interquartile range)

*Abbreviations* *FMA* Fugl-Meyer assessment

^#^ *p*<0.05 for intragroup comparison

## Discussion

This randomized double-blinded controlled trial is the first study to compare the immediate effects of different tDCS placements on dual task walking performance and corticomotor activity in individuals with stroke. In this study, we found that one session of bilateral tDCS and cathodal tDCS for 20 min improved walking speed during both cognitive dual task walking and motor dual task walking as compared with the sham tDCS. Moreover, the increased walking speed after bilateral tDCS and cathodal tDCS also significantly more than the anodal tDCS during motor dual task walking. Regarding the brain activity, both bilateral and cathodal tDCS significantly increased the contralesional corticomotor inhibition indicated by silent period compared with sham tDCS. The anodal tDCS to lesioned hemisphere, however, did not seem to exert significant immediate effects on modulating corticomotor activity and walking performance in our participants with chronic stroke.

There is limited investigation regarding the tDCS effects on dual task walking performance although the dual task walking ability is necessary for daily activities [5]. In this study, the cathodal and bilateral tDCS improved dual task walking ability which is most challenging to stroke patients. Liu et al. reported that dual task training for 12 sessions resulted in 2~12% improvements of dual task walking speed in chronic stroke [32]. In current study, the dual task walking speed could be improved by 12~22% immediately after one session bilateral tDCS or cathodal tDCS in chronic stroke. The participants’ walking speed before intervention was comparable in Liu et al’s study (60.3±17.2 cm/sec) and present study (61.04±20.9 cm/sec). Therefore, bilateral tDCS and cathodal tDCS seem to be an effective intervention to immediately improve both cognitive and motor dual task walking ability for individuals with chronic stroke, however, the accumulative effects on dual task walking performance need to be further documented. Moreover, for single walking performance, Tahtis et al. reported single session of bilateral tDCS improved the timed up and go test in their subacute stroke participants [12]. Our results also showed that bilateral tDCS improved the single walking speed in chronic stroke patients, while cathodal tDCS only exerted a trend of improvement (*p*= 0.09). Recently, Seamon et al. demonstrated no significant effect after single session of anodal, cathodal, or bilateral tDCS compared with sham tDCS on gait performance in their double-blind randomized cross-over trial in participants with chronic stroke [14]. Participants in Seamon et al’s study could be categorized as the community ambulators (averaged walking speed: 0.82 m/s) while our participants could be categorized as the limited community ambulatory (averaged walking speed: 0.61 m/s) [33]. We thus speculate the effects of tDCS may be influenced by the participant’s walking ability.

In present study, we found that the improvement in CDT and MDT walking speed paralleled to the increase in SP of contralesional hemisphere. According to a meta-analysis, the suppression of contralesional M1 improved upper extremity motor function in chronic stroke [34]. The interhemispheric interaction has been suggested by a meta-analysis which indicated the decreased contralesional excitability with increased ipsilesional excitability by low frequency rTMS applied to contralesional hemisphere [35]. We thus speculate that the effects of cathodal and bilateral tDCS in our study may be due to the inhibitory effects of cathodal stimulation as observed from the low frequency rTMS. Zimerman *et al*. found that cathodal tDCS enhanced the training effects and modulated intracortical inhibition in ipsilesional and contralesional M1[15]. Stagg et al., showed cathodal tDCS improved UE motor performance with increased activity in ipsilesional M1 as compared with sham tDCS [36]. Recently, Kuo et al. showed bilateral tDCS immediately increased cortical excitability and decreased transcallosal inhibition in ipsilesional M1, with a decrease in excitability and an increase in transcallosal inhibition in contralesional M1 [37]. Our results of cathodal and bilateral tDCS on corticomotor activity in contralesional M1 were in line with Kuo et al.’s study. Moreover, in addition to increasing the inhibition, reducing the excitatory transmission has also been proposed for the inhibitory effects [38]. Therefore, the possible mechanisms of inhibitory effect by cathodal stimulation need further elucidation.

Our results indicated only the SP increased after cathodal and bilateral tDCS (Fig. 3), but not the SICI. Therefore, the GABA_B-_ergic system may play a role in cathodal stimulation mechanism. The present study was the first study to document the modulation of contralesional hemisphere and improvement of walking performance immediately after tDCS. A recent meta-analysis indicated that SICI was different between ipsilesional and contralesional hemisphere in early stroke, but not in chronic stroke. However, the SP was different at both stages after stroke [39]. Thus, we suggested that the SICI may not be sensitive enough to document the intracortical inhibition in chronic stroke.

There was no significant walking improvement after the anodal tDCS in this study. Previous studies have reported that single session anodal tDCS did not improve gait performance [13], nor the 10 sessions program of anodal tDCS and conventional physical therapy [40]. Park et al. also demonstrated that 12 sessions of anodal tDCS combining exercise did not improve gait velocity when compared with sham tDCS with exercise [41] which were consistent with present study. On the other hand, a meta-analysis showed that tDCS combined with other therapies did not significantly improve gait performance [42]. However, it should be noted that six of the seven included studies investigated the anodal tDCS stimulation. It has been suggested that the resting motor threshold of the ipsilesional hemisphere increased after stroke [39]. Therefore, the maximal 2 mA output of current tDCS may not be sufficient to immediately modulate the corticomotor activity to enhance motor performance by anodal tDCS directly to ipsilesional hemisphere. Such ineffective modulation of the anodal DCS has also been reported previously [43]. However, Madhavan *et al*. found anodal tDCS improved motor control of the ankle and enhanced cortical excitability in ipsilesional M1, while the MEP in ipsilesional hemisphere could be induced in most of their participants before intervention [44]. Stagg et al. also demonstrated increased ipsilesional M1 activity after anodal tDCS in a study with greater than 40% patients having massive cortical stroke [36]. However, there is only 8% patients with cortical stroke in present study. Taking together, the beneficial effects of anodal tDCS may depend at least on the resting motor threshold of the lesioned hemisphere and the lesional location.

We noted the LE motor control as indicated by FMA did improve after one-session of cathodal and bilateral tDCS, but without significant difference as comparing the sham group. By contrast, O'Shea et al. and Fleming et al. found significant upper limb improvements following one session of tDCS [45, 46]. However, Chang et al. demonstrated the significant effects in FMA-LE after 10 sessions tDCS combined physical therapy compared with sham group [40]. Therefore, FMA of lower extremity may not be as a sensitive measurement as walking performance, particularly as the dual task walking to document the immediate effect after single session of tDCS.

## Limitations

There are some limitations of this study. First, only assessing the immediate effects in the present study should be noted. Ojardias et al. demonstrated nonsignificant effects during stimulation, however, with the significant effects after 1 h after tDCS stimulation [47]. Second, we only documented the activity of contralesional hemisphere by TMS, therefore, our results are insufficient to explain the effects of tDCS stimulation on the interactions between two hemispheres. Third, it has been reported that lesion location and post-onset duration may influence the effects of tDCS [12, 48, 49]. In present study, we recruited people with both cortical and subcortical chronic stroke (Table S1), therefore, it should be careful to generate our study results.

## Conclusion

This randomized double-blinded controlled trial is the first study to demonstrate that bilateral tDCS and cathodal tDCS exerted immediate effects on dual task walking performance due to at least decrease the contralesional corticomotor activity in chronic stroke patients.

## Supplementary Information

**Additional file 1:** **Supplemental Table 1.** Demographic characteristics of individual stroke participants (*n* = 48).

## Data Availability

The datasets used and/or analyzed for this study are available from the corresponding author on reasonable request.

## Abbreviations

CDT: Cognitive dual task
CV: Coefficients of variation
DTC: Dual-task cost
EEG: Electroencephalography
ES: Effect size
EMG: Electromyographic
FMA: Fugl-Meyer assessment scale
GABA: Gamma-aminobutyric acid
LE: Lower extremity
MDT: Motor dual task
MEP: Motor evoked potentials
M1: Primary motor area
MMSE: Mini-mental state examination
NIBS: Non-invasive brain stimulation
RMT: Resting motor threshold
SP: Silent period
SICI: Short interval intracortical inhibition
TA: Tibialis anterior
tDCS: Transcranial direct current stimulation
TMS: Transcranial magnetic stimulation
