# Correction: Comparing different montages of transcranial direct current stimulation on dual-task walking and cortical activity in chronic stroke: double-blinded randomized controlled trial

**DOI:** 10.1186/s12883-024-03996-3

**Published:** 2025-01-07

**Authors:** Pei‑Ling Wong, Yea‑Ru Yang, Shun‑Chang Tang, Shih-Fong Huang, Ray‑Yau Wang

**Affiliations:** 1 Department of Physical Therapy and Assistive Technology, National Yang Ming Chiao Tung University, Taipei, Taiwan ROC; 2 Division of Nerve Repair‑ Department of Neurosurgery, Taipei Veterans General Hospital, Taipei, Taiwan ROC

**Correction: BMC Neurol 22**,** 119 (2022)**.

10.1186/s12883-022-02644-y.

Following publication of the original article [1], the authors identified an error in the author name of Shih‑Fong Huang.

The incorrect author name is: Shi-Fong Huang.

The correct author name is: Shih-Fong Huang.

The original article [1] has been updated.
