# Supplementary material for: Comparing different montages of transcranial direct current stimulation on dual-task walking and cortical activity in chronic stroke: double-blinded randomized controlled trial
Source: BMC Neurol. 2022 Mar 25;22:119. doi: 10.1186/s12883-022-02644-y (PMC8951706; doi:10.1186/s12883-022-02644-y)
Supplement: Supplementary file 1 — Additional file 1: Supplemental Table 1. Demographic characteristics of individual stroke participants (n = 48). [file 12883_2022_2644_MOESM1_ESM.docx]

| **Supplemental table 1.** Demographic characteristics of individual stroke participants (n=48). |
| --- |
| \| Patient \| Group \| Age \| Sex \| Type of stroke(H/I) \| Lesion hemisphere \| Post stroke period (months) \| MMSE \| Lesion site \| \| --- \| --- \| --- \| --- \| --- \| --- \| --- \| --- \| --- \| \| 1 \| A \| 60.6 \| M \| H \| Right \| 89.4 \| 28 \| internal capsule \| \| 2 \| A \| 46.8 \| M \| H \| Right \| 86.5 \| 30 \| basal ganglia \| \| 3 \| A \| 73.7 \| M \| H \| Right \| 222.5 \| 29 \| brain stem \| \| 4 \| A \| 65.9 \| M \| I \| Right \| 82.1 \| 29 \| insula \| \| 5 \| A \| 46.7 \| M \| H \| Right \| 49 \| 27 \| thalamus \| \| 6 \| A \| 65.8 \| M \| I \| Left \| 64 \| 30 \| putamen, thalamus \| \| 7 \| A \| 58.9 \| M \| H \| Right \| 111.4 \| 30 \| thalamus \| \| 8 \| A \| 51.9 \| M \| I \| Right \| 68.7 \| 30 \| thalamic, parietal lobe \| \| 9 \| A \| 44.8 \| F \| H \| Right \| 80.1 \| 30 \| temporal lobe \| \| 10 \| A \| 34.1 \| M \| I \| Right \| 10.8 \| 29 \| thalamus \| \| 11 \| A \| 53.4 \| M \| I \| Left \| 8 \| 29 \| basal ganglia \| \| 12 \| A \| 40.0 \| F \| I \| 1Right \| 8 \| 29 \| corona radiata \| \| 13 \| B \| 59.3 \| M \| I \| Right \| 50.4 \| 29 \| brain stem \| \| 14 \| B \| 33.4 \| M \| H \| Right \| 71.9 \| 29 \| basal ganglia \| \| 15 \| B \| 71.5 \| M \| I \| Left \| 71.9 \| 25 \| putamen \| \| 16 \| B \| 53.1 \| M \| H \| Right \| 90.7 \| 30 \| frontal lobe \| \| 17 \| B \| 38.0 \| F \| I \| Left \| 71.1 \| 25 \| basal ganglia \| \| 18 \| B \| 66.8 \| M \| I \| Right \| 92.3 \| 30 \| corona radiata \| \| 19 \| B \| 47.8 \| F \| I \| Right \| 106.7 \| 30 \| subcortical regions \| \| 20 \| B \| 59.8 \| M \| H \| Right \| 32.9 \| 29 \| occipital 3Lobe \| \| 21 \| B \| 44.7 \| M \| H \| Right \| 51.9 \| 30 \| putamen, thalamus \| \| 22 \| B \| 67.0 \| M \| I \| Right \| 62 \| 27 \| thalamus \| \| 23 \| B \| 41.8 \| F \| I \| Left \| 29.4 \| 29 \| internal capsule \| \| 24 \| B \| 50.5 \| M \| I \| Right \| 17 \| 30 \| pons, basal ganglia \| \| 25 \| C \| 57.0 \| F \| H \| Left \| 99.3 \| 28 \| corona radiata \| \| 26 \| C \| 38.1 \| M \| H \| Left \| 42 \| 24 \| putamen \| \| 27 \| C \| 71.4 \| M \| I \| Right \| 82.2 \| 25 \| parietal lobe, basal ganglia \| \| 28 \| C \| 37.8 \| M \| H \| Left \| 14.1 \| 30 \| basal ganglia \| \| 29 \| C \| 54.9 \| M \| I \| Left \| 19 \| 29 \| pons(pontomedullary) \| \| 30 \| C \| 71.6 \| F \| I \| Left \| 23.1 \| 29 \| corona radiata \| \| 31 \| C \| 63.5 \| M \| I \| Left \| 48.2 \| 30 \| pons \| \| 32 \| C \| 61.2 \| M \| H \| Right \| 23.7 \| 28 \| basal ganglia \| \| 33 \| C \| 53.0 \| M \| I \| Left \| 7.2 \| 30 \| brain stem \| \| 34 \| C \| 51.1 \| M \| H \| Right \| 133 \| 30 \| basal ganglia \| \| 35 \| C \| 67.9 \| M \| H \| Right \| 135 \| 30 \| internal capsule \| \| 36 \| C \| 62.4 \| M \| H \| Right \| 106.9 \| 29 \| subcortical regions \| \| 37 \| D \| 67.6 \| M \| I \| Left \| 95 \| 28 \| internal capsule \| \| 38 \| D \| 63.5 \| M \| H \| Right \| 88.4 \| 28 \| corona radiata \| \| 39 \| D \| 55.9 \| M \| I \| Left \| 55 \| 30 \| putamen \| \| 40 \| D \| 60.6 \| F \| H \| Left \| 115.9 \| 27 \| putamen, thalamus \| \| 41 \| D \| 60.0 \| M \| H \| Left \| 66.2 \| 26 \| corona radiata \| \| 42 \| D \| 45.0 \| M \| I \| Right \| 141 \| 30 \| basal ganglia \| \| 43 \| D \| 56.2 \| M \| H \| Left \| 53.2 \| 25 \| internal capsule, frontal lobe \| \| 44 \| D \| 44.6 \| F \| H \| Left \| 38.5 \| 29 \| putamen \| \| 45 \| D \| 71.1 \| M \| I \| Right \| 24 \| 28 \| frontal lobe \| \| 46 \| D \| 58.3 \| F \| H \| Left \| 24 \| 30 \| putamen \| \| 47 \| D \| 49.6 \| M \| H \| Left \| 9 \| 26 \| thalamus, lentiform nucleus \| \| 48 \| D \| 29.1 \| M \| H \| Left \| 9 \| 30 \| putamen \| |
| Abbreviations: M, Male; F, Female; H, hemorrhagic; I, Ischemic. MMSE, mini-mental state examination;  Group A, anodal tDCS; group B, bilateral tDCS; group C, cathodal tDCS; group D, sham tDCS. |
